# Epigenetic Control of Innate Immunity: Consequences of Acute Respiratory Virus Infection

**DOI:** 10.3390/v16020197

**Published:** 2024-01-27

**Authors:** Rivka Bella Lefkowitz, Clare M. Miller, Juan David Martinez-Caballero, Irene Ramos

**Affiliations:** 1 Department of Neurology, Icahn School of Medicine at Mount Sinai, New York, NY 10029, USA; rivka.rabaev@mssm.edu (R.B.L.); clare.miller@mssm.edu (C.M.M.);; 2 Precision Immunology Institute, Icahn School of Medicine at Mount Sinai, New York, NY 10029, USA; 3 Graduate School of Biomedical Sciences, Icahn School of Medicine at Mount Sinai, New York, NY 10029, USA

**Keywords:** innate immunity, acute infection, virus, DNA methylation, histones, epigenetics

## Abstract

Infections caused by acute respiratory viruses induce a systemic innate immune response, which can be measured by the increased levels of expression of inflammatory genes in immune cells. There is growing evidence that these acute viral infections, alongside transient transcriptomic responses, induce epigenetic remodeling as part of the immune response, such as DNA methylation and histone modifications, which might persist after the infection is cleared. In this article, we first review the primary mechanisms of epigenetic remodeling in the context of innate immunity and inflammation, which are crucial for the regulation of the immune response to viral infections. Next, we delve into the existing knowledge concerning the impact of respiratory virus infections on the epigenome, focusing on Severe Acute Respiratory Syndrome Coronavirus 2 (SARS-CoV-2), Influenza A Virus (IAV), and Respiratory Syncytial Virus (RSV). Finally, we offer perspectives on the potential consequences of virus-induced epigenetic remodeling and open questions in the field that are currently under investigation.

## 1. Introduction

When viruses infect an organism, components of the virus are detected in the cells, activating a broad range of innate immune pathways to counteract those infections. Viral nucleic acids and other Pathogen-Associated Molecular Patterns (PAMPs) are sensed by surface or endocytic Pattern Recognition Receptors (PRRs) in infected cells, such as Toll-like receptors (e.g., TLR3, TLR7, and TLR9) or by cytoplasmic receptors such as the RNA sensors Retinoic Acid-Inducible Gene-I (RIG-I), Melanoma-Differentiation-Associated gene 5 (MDA5), Nucleotide-binding domain and Leucine-rich Repeat containing (NLRs) [1,2] or DNA sensors such DNA-dependent Activator of Interferon-regulatory factors (DAI), Absent In Melanoma (AIM) 2, or cyclic GMP–AMP Synthase (cGAS), leading to downstream signaling cascades involving the effector Stimulator of Interferon Genes (STING) [3]. Importantly, receptor sensing is not only PAMP-specific but also cell-type specific, which is determined mostly by the levels of expression of those receptors in different cell types. Sensing through these different mechanisms leads to the activation of multiple transcriptional programs, including those leading to the production of type I and type III Interferon (IFN) genes and NF-kappaB-driven cytokines [2,4,5]. Through paracrine signaling, type I and type III IFN activate the transcription of IFN-stimulated genes (ISGs), which primarily leads to antiviral immunity.

In addition to regulation through transcriptional [6,7], post-transcriptional, and [8,9] post-translational [10,11,12] mechanisms, epigenetic events have a critical impact on the modulation of innate immunity. The epigenetic state of the cells before stimuli greatly defines and shapes their transcriptional response to infection [13]. However, the epigenome is also altered upon viral infection, which could contribute to innate immune response to subsequent stimuli, adaptive immunity, or possibly elicit autoimmune processes. Thus, epigenetic remodeling is a dynamic system shaped by external exposures, including pathogens, that contribute to the modulation of the immune response to infection. In this article, we lay the groundwork by comprehensively describing the epigenetic mechanisms governing innate immunity. Subsequently, we examine the literature to scrutinize how respiratory virus infections remodel the epigenetic landscape, offer insights into current knowledge gaps, and propose intriguing questions for future research investigations. The current knowledge on this subject will contribute to paving the road towards a better understanding of the possible consequences of these epigenetic events and the identification of therapies to control potential detrimental effects.

## 2. Mechanisms of Epigenetic Regulation in Innate Immunity

Most of the components of the innate immune response to infection are not constitutively expressed or are expressed at low levels at baseline. Thus, its expression is induced upon the detection of PAMPs and the subsequent activation of signaling cascades, as well as the activation of transcription factors to initiate gene expression. However, the epigenetic properties of regulatory regions of those genes at the time of innate immune detection are critical to the levels of innate immune activation. These epigenetic processes, including DNA methylation, histone modifications, and chromatin structure, are highly complementary and work together to allow for activation or repression of gene transcription. Cell lineages are epigenetically determined during development, which in part also determines the cellular specificity of the transcriptional profile upon innate immune stimulation. In this section, we provide an overview of the current understanding of the mechanisms of epigenetic regulation and their known roles in the context of innate immunity.

### 2.1. DNA Methylation

DNA methylation and demethylation are complex processes that greatly contribute to the control of gene expression. DNA methylation consists of the addition of a methyl group onto carbon 5 of a cytosine base, resulting in the formation of 5-methylcytosine (5mC). The main functions of 5mC involve X chromosome inactivation, genomic imprinting, and the regulation of transcription [14]. In the proximity of promoter regions, DNA methylation prevents transcription by blocking the binding of transcription factors. DNA demethylation, on the other hand, restores the naked DNA form through a series of oxidative reactions combined with Base Excision Repair (BER), thus unlocking the genome [14].

The main actors involved in the epigenetic regulation of DNA can be broken down into three categories of enzymes: “writers”, “readers”, and “erasers”. Together, they orchestrate the epigenetic changes that allow cells to exhibit fine-tuned responses to environmental stimuli [15]. The “writers” are enzymes responsible for the addition of methyl groups to cytosine residues. The enzymes that catalyze this reaction belong to the DNA Methyltransferase (DNMT) family. DNMT1, known for its role in methylation maintenance, is responsible for most of the DNA methylation in human and mouse genomes. It preferentially localizes to hemi-methylated sites on a newly formed DNA strand during DNA replication and methylates the DNA to precisely copy the previous methylation pattern. DNMT3a and DNMT3b, on the other hand, are responsible for de novo methylations during cell replication [15]. The “readers” continue the process of methylation by recognizing and interpreting epigenetic markers. DNA methylation is recognized by three main classes of reader proteins: Methylated DNA Binding domain proteins (MBDs), Ubiquitin-like containing PHD and RING Finger proteins (UHRFs), and zinc finger-like proteins [15]. MBDs are attracted to the methylated areas of the genome and induce histone modifications, which result in a chromatin configuration that makes the DNA inaccessible [16,17]. They also contain Transcriptional Repression Domains (TRDs), which allow them to form complexes with other repressors and strengthen transcription repression [15]. UHRFs maintain DNA methylation through the tethering of DNMT1 to the binding site and play a crucial role in localizing DNMT1 to hemi-methylated regions [18]. Both MBDs and UHRFs may play a role in recruiting DNMT1s to existing hemi-methylated sites [19]. The “erasers” are responsible for the demethylation of the genome, a process that restores genes to their unmodified state and can occur actively or passively. Active demethylation is facilitated by the Ten-Eleven Translocation (TET) proteins and involves the restoration of 5mc to its original un-methylated form through the iterative oxidation of 5mC to 5-hydroxymethylcytosine (5hmC), 5-formylcytosine (5fC) and 5-carboxylcytosine (5caC) (reviewed by [20]). These modified cytosines can be recognized by Thymine DNA Glycosylase (TDG) and replaced with an unmodified cytosine through BER. Passive demethylation occurs in dividing cells in the absence of the methylation maintenance performed by DNMT1 [14].

CpG islands (CGIs) are CpG-rich regions in the genome, which are mostly found at sites of transcription initiation [21]. CGIs are frequently unmethylated, although there is diversity in the length of the CGIs and their methylation levels [22]. The chromatin structure in promoters with CGIs is, in general, transcriptionally permissive. RNA Polymerase II (RNA PolII) is bound at many CGI promoters of Lipopolysaccharide (LPS)-inducible genes in unstimulated conditions in macrophages, and transcription activation is controlled at the transcriptional elongation and mRNA processing steps [23]. The silencing of CGI promoter occurs through DNA methylation, either by directly inhibiting transcription factor binding or by the recruitment of MBDs [24].

There is increasing evidence in the literature that changes in DNA methylation govern many aspects of the innate immune response. Ex vivo studies using human primary immune cells have shown that differentiation of monocytes to Dendritic Cells (DCs) and DC maturation are associated with dynamic DNA methylation patterns and changes in the expression of DNMT1, DNMT3a, DNMT3b and TET2 [25]. In a recent study, it was found that mutations and silencing in DNMT3a and TET2 affected mitochondria integrity, leading to increased type-I IFN responses in human monocyte-derived macrophages as well as mouse bone-marrow-derived macrophages [26]. However, in vivo studies developed by Li et al. [27] showed that the knocking down of Dnmt3a selectively in myeloid cells in vivo decreased the production of type I IFN after exposure to multiple TLR ligands and viral challenge, and that those mice presented higher mortality when challenged with Vesicular Stomatitis Virus (VSV) than WT mice. Interestingly, in this case, there was not a direct effect of Dnmt3a in the methylation of the Ifnb1 promoter gene, but its absence resulted in decreased methylation of Hdac9, which led to an increase in protein expression. HDAC9 did not act by directly regulating gene expression either, but by enhancing the activity of TBK1, resulting in increased phosphorylation of IRF3 [27]. Another study found a role for DNMT1 in promoting the induction of LPS-induced pro-inflammatory cytokines in macrophages via the hypermethylation of the SOCS1 promoter, which is a negative regulator of cytokine response [28]. TET2 has been shown to regulate the differentiation of M1 macrophages [29], and the loss of TET2 expression results in increased LPS-induced inflammation [30]. In addition to modulating cytokine expression through active demethylation, TET2 also represses IL-6 through the recruitment of the histone deacetylase HDAC2 in innate myeloid cells [31].

### 2.2. Histone Modifications

Histones H2A, H2B, H3, and H4 are key in maintaining the chromatin structure. Nucleosomes are formed by an octamer of histones that are wrapped into DNA fibers. Histone tails protruding from the nucleosomes are modified through post-translational modifications of lysine or arginine residues. Multiple post-translational modifications of histones have been associated with the regulation of transcription, including methylation, acetylation, ubiquitination, ADP-ribosylation, sumoylation, and phosphorylation, which have been extensively reviewed [32]. Methylation and acetylation are the most studied histone modifications. The methylation of lysine or arginine residues of histone proteins is mediated by Histone Methyltransferases (HMTs). Acetylation is mediated by Histone Acetyltransferases (HATs), and Histone Deacetylases (HDACs) promote the removal of the acetyl group [33]. Some of the post-translational modifications of histones are associated with active transcription, and the chromatin affected by these marks is referred to as euchromatin. Some examples are H3K4me3, H3K4me2, H3K56ac, and H4K16ac (reviewed by [32]). On the other hand, repressive changes are often referred to as heterochromatin modifications. H3K9me, H3K27me [32], and H3K4me1 [34] are examples of histone marks that result in the inhibition of gene transcription.

Both H3K27me3 and H3K9me marks were found to act as regulators of inflammation. H3K27me3 can be demethylated by an LPS-induced demethylase named Jumonji C (JmjC) domain protein 3. Jmjd3 binds to the Polycomb group (PcG, which are complexes of proteins with important roles in epigenetic regulation) target genes and regulates H3K27me3 levels and, therefore, the transcription of those genes [35]. Research conducted by the same group revealed that, in unstimulated cells, H3K9 is heavily methylated in subsets of promoters of inflammatory genes. However, upon activation, methylation decreases, showing a correlation with RNA PolII recruitment, and is then restored post-stimulation [36]. Another example of the regulation of inflammation through the methylation of histones is the role of the SET and MYND domain-containing 2 (Smyd2) methyltransferase in macrophages, which specifically facilitates H3K36 dimethylation at Tnf and Il6 promoters, suppressing their transcription [37].

Histone modifications have been shown to contribute to the tight regulation of the IFNB1 promoter during virus infection. The IFNB1 enhanceosome, whose assembly is necessary for transcriptional activation, consists of multiple factors recruited to the promoter, including NF-kappaB, ATF-2/c-jun, IRF3, and IRF7 [38,39]. After viral infection, the hyperacetylation of H3 and H4, which is correlated with active chromatin, was found in the promoter and 5′ end of the IFNB1 gene [40]. Disrupting the recruitment of HATs (CBP/p300) to the enhanceosome led to a decrease in IFNB1 gene expression [40]. Therefore, H3 and H4 acetylation play a critical role in regulating type I IFN response upon viral infection.

The downstream response to type I IFN, which results in the activation of the expression of hundreds of ISGs, is also modulated by histone modifications. It was shown that, upon IFN-alpha signaling, the transcription factor STAT2 recruits an acetyltransferase (GCN5) and H3 is acetylated in the ISG promoters [41]. Bromodomain-containing protein 4 (BRD4) is an important player in this process since, through binding to acetylated histones in ISGs, it coordinates the recruitment of the pause release factor P-TEFb to initiate elongation [42]. Type I IFN also upregulates Setdb2, a lysine methyltransferase that trimethylates H3K9, resulting in a reduction in the expression levels of several ISGs, including Mx1 and Isg15 [43].

The impact of histone modifications in regulating innate immunity has been highlighted by the important finding that some histone changes induced upon immune activation in inflammatory genes persist for long periods of time, providing innate immune memory and resulting in modified responses upon subsequent innate immune activation. This type of immune memory might result in protection against heterologous pathogens, which has been referred to as trained immunity [44], and research in this area has been actively developed during the last 13 years (review by [45]). Interestingly, trained immunity-mediated heterologous protection can be transmitted through generations [46]. Epigenetic reprogramming, however, can also result in innate immune tolerance, characterized by a decreased immune response against a secondary infection [47].

One classic example of the trained immunity phenomenon is the protection conferred by the BCG vaccine against Candida Albicans and other infections [48,49,50]. Mechanistically, it was found that, after BCG vaccination, there is increased H4K3me levels in regulatory regions of pro-inflammatory genes such as TNF-alpha or IL-6 in monocytes, which results in an increased ability of those monocytes to respond to subsequent heterologous exposures [49]. BCG-induced training of monocytes is also accompanied by decreased levels of the repressor mark in those promoters, and those epigenetic changes are associated with changes in glucose metabolism [51]. BCG was also found to induce trained immunity in natural killer (NK) cells, as shown by increased levels of cytokines upon heterologous ex vivo stimulation of NK cells from BCG-vaccinated healthy subjects with respect to prior vaccination [52].

In murine macrophages, it was shown that LPS stimulation induces either tolerance or increased responses in different groups of genes upon re-stimulation [47]. In both groups of genes, promoters showed increased H4 acetylation and H3K4 trimethylation upon stimulation. However, promoters of the group of genes in the “tolerance” group present lower levels of histone acetylation upon re-stimulation and lower chromatin accessibility, while those with increased responses become more H4 acetylated and accessible [47]. Therefore, TLR-4-mediated stimulation of macrophages induces memory or tolerance in different genes, which is regulated at the epigenetic level through H4 acetylation.

### 2.3. Chromatin Structure and Accessibility

Chromatin structure plays a critical role in the development of cell types by determining the expression of cell-specific factors, such as PRRs or transcription factors, in accordance with their main functions in the immune response [53]. In addition, chromatin remodeling is an essential process that controls transcription, as well as DNA replication and repair. These processes allow accessibility to DNA interacting proteins to execute their functions, and therefore it has a highly important role in the regulation of gene expression upon immune stimulation. There is a broad number of proteins that regulate chromatin structure and accessibility. ATP-dependent chromatin remodelers have a crucial role in repositioning or modifying nucleosomes. There are four families of chromatin remodelers in eukaryotic cells (reviewed by [54,55]), and all of them have roles in modulating transcription, in some cases, with known functions in innate immunity:

(i) The switch/sucrose non-fermentable (SWI/SNF) family, which includes the Remodeling the Structure of Chromatin (RSC) and SWI/SNF complexes. SWI/SNF complexes weaken the interactions between histones and DNA, therefore increasing accessibility to transcription factor binding, and work in a gene- and factor-specific manner [56,57]. SWI/SNF complexes were found to be important in the modulation of innate immunity, in particular in the regulation of TLR-induced (TLR-2, TLR-3, and TLR-4) or cytokine-induced responses [58]. Some genes are dependent on SWI/SNF complexes for their induction. Most SWI/SNF-dependent genes contain promoters with low CpG content, while most SWI/SNF-independent genes contain promoters with CGIs [53,58]. CGIs promoters have traits of active chromatin before stimulation: low nucleosome occupancy and high levels of H3K4me3. This suggests that they do not require SWI/SNF because they are already in an adequate conformation for activation upon stimulation. Studies have shown that the requirement of SWI/SNF complexes for the expression of LPS-induced genes is highly variable and seems to be associated with their role as primary response genes (expression induced by transcription factors activated by post-translational mechanisms in response to stimulus) or secondary responses (synthesis of at least one protein from the primary response needed for gene expression) [58,59]. Thus, most primary response genes are SWI/SNF-independent, while most secondary response genes are SWI/SNF-dependent [53,58].
(ii) The Imitation Switch (ISWI) complexes contribute to the maturation of initial histone–DNA complexes or pre-nucleosomes. They are involved in transcriptional regulation, but also DNA repair and recombination (reviewed by [60]). The ISWI family is composed of seven different subfamilies (ACF, CHRAC, NoRC, WICH, RSF, NURF and CERF). The ACF and CHRAC complexes promote repressive chromatin and, therefore, reduce gene transcription [61]. The Nucleosome Remodeling Factor (NURF) contributes to the regulation of innate immunity in Drosophila. NURF modifies the chromatin structure by sliding nucleosomes, and it was found to be a co-repressor of a set of JAK/STAT target genes [62].
(iii) Cromodomain Helicase DNA-binding (CHD) complexes can contribute to the activation or repression of gene expression [63]. The members of this family present an N-terminal double chromodomain, which mediates association with chromatin through direct binding to the methylated lysins on H3 [64], RNA [65] or nucleosomal DNA [66]. The complex CHD4 (Mi-2 beta)/NuRD has been shown to have a role in regulating the expression of NF-kappaB-induced genes and has an opposite role to SWI/SNF complexes upon the LPS stimulation of macrophages. The CHD4/NuRD complex includes histone deacetylase factors. It is recruited upon LPS activation with SWI/SNF to secondary or late primary response genes, acting antagonistically to inhibit the activation of those genes [59].
(iv) The INOsitol-requiring 80 (INO80) family includes three complexes in mammals: INO80, Snf2-related CREBBP activator protein (SRCAP), and p400 complexes. SRCAP and p400 complexes form the SWR1 class. They are involved in DNA repair, DNA replication and the regulation of transcription [67,68]. The members of this family can regulate the dynamics of histone variants by catalyzing ATP-dependent histone dimer exchange reactions. Members of the INO80 and the SWR1 enzymes work together to regulate the localization of H2A and H2A.Z variant histones [68,69]. This might have important implications in the dynamics of cellular functions. For example, INO80, through the eviction of H2A.Z from the promoter leads to a reduction in the transcription of genes involved in autophagy [70].

In addition to chromatin remodelers, transcription factors also might promote changes in chromatin structure, particularly during innate immune stimulation. For transcription to take place, after specific transcription factors interact with the promoter DNA, the general transcription machinery needs to be recruited, such as the Mediator complex, General Transcription Factors (GTFs), and RNA PolII. The recruitment of specific transcription factors can sequentially change the chromatin structure, allowing for further recruitment of the components of the general transcription machinery and the activation of transcription. The IFNB1 promoter represents an example of this concept. As indicated above, the IFNB1 enhanceosome is assembled through the recruitment of multiple transcription factors [38,39]. The assembly of the enhanceosome occurs in a nucleosome-free enhancer region of the gene and results in the modification of a nucleosome, initially masking the TATA box and the start site. Then, the GCN5 complex is recruited to the enhanceosome, leading to the acetylation of the two adjacent nucleosomes, followed by the recruitment of the CBP-Poll II complex. Acetylation of the nucleosomes facilitates the recruitment of the SWI/SNF complex through interactions with CBP, and the SWI/SNF complex remodels the chromatin, allowing for binding of TFIID to the TATA box and leading to transcription initiation [71].

### 2.4. Non-Coding RNAs

Non-coding RNAs also play an important role in the regulation of innate immunity at the epigenetic level. Long non-coding RNAs (lncRNAs) can attract DNA or RNA-binding proteins to regulatory regions to induce or prevent histone modifications or limit the binding of transcription factors to the promoter region [72]. LncRNAs such as NEAT1 and MALAT1 have been associated with the modulation of innate immune responses and inflammation [73,74,75]. Research on various viral infections identified specific micro RNAs (miRNAs) that target viral genes or modulate host immune responses. For example, miRNAs might suppress transcription by recruiting other proteins to enhance histone modifications or DNA methylation [76]. Specific miRNAs, such as miR-155, exhibit altered expression patterns during viral infections, influencing the expression of immune-related genes [73,77].

## 3. Effects of Respiratory Virus Infection in the Epigenetic Modulation of Innate Immunity

The epigenetic state of inducible genes plays a major role in modulating the immune response by allowing activation or by repressing specific immune genes upon innate immune stimulation. Importantly, there is increased evidence that exposure to viral infection also alters the epigenetic makeup of the immune cells, which might have important implications in the subsequent response to infection or to other exposures, as well as in the overall health state. In this section, we review the literature on how viral exposure, specifically SARS-CoV-2, influenza A virus (IAV), or RSV infections, induces epigenetic changes in innate immune cells or in infected cells in our organism.

### 3.1. Evidence of Epigenetic Immune Regulation after Acute Respiratory Virus Infection from Human Studies

The recent SARS-CoV-2 pandemic has led to multiple studies analyzing the epigenetic profile of the blood of individuals previously infected by SARS-CoV-2 (Table 1). Using samples from a large cohort of young recruits as part of the COVID-19 Health Action Response for Marines (CHARM) study, we found that the DNA methylation profile in blood cells after infection is modified with respect to the pre-infection profile, and these changes persist for at least several weeks [78]. This signature was characterized by a high proportion of hypomethylated regions in proximity to ISGs. Additionally, we found a large overlap with previously reported signatures associated with autoimmune diseases such as systemic lupus erythematosus or multiple sclerosis [78], indicating a potential dysregulation of the innate immune system.

A different study that analyzed epigenetic response in eight convalescent individuals (4–12 weeks after recovery) in blood cells using single-cell ATAC-seq assays found different chromatin accessibility profiles as compared to healthy individuals, particularly in CD14^+^ and CD16^+^ monocytes [79]. This profile was characterized by an increase in accessibility of IRF1, IRF3 and IRF8 transcription factor motifs. PBMCs from individuals convalescing from COVID-19 secreted higher levels of IL-1β and IL-6 than those from the healthy donors upon challenge with a different virus [79]. Another study used single-cell RNA-seq, single-cell ATAC-seq and DNA methylation analysis to characterize the epigenetic responses in individuals who had recovered from COVID-19 [80]. Here, minor epigenetic differences were found between the controls and convalescence participants. Limited differences in DNA methylation were identified in the two groups, which were mapped to monocytes and CD4^+^ T cells. Interestingly, a recent study found long-term post-COVID-19 epigenetic signatures by analyzing chromatin accessibility in Hematopoietic Stem and Progenitor Cells (HSPC), which greatly resembled those found in CD14^+^ monocytes, contributing to our understanding of the maintenance of those changes [81].

Overall, these studies indicate that SARS-CoV-2 acute respiratory viral infections promote long-term epigenetic changes in the immune system. However, the post-infection consequences of these changes in terms of subsequent response to immune perturbations, accelerated aging, or persistent symptoms are not known. Early after the emergence of SARS-CoV-2, it became evident that some individuals suffering from COVID-19 experience persistent symptoms, collectively termed Post-Acute Sequelae of SARS-CoV-2 infection (PASC) or “long COVID” [82]. It is possible that virus-induced epigenetic modification contributes to the development of PASC, and this hypothesis is currently under investigation.

RSV infections in children have also been associated with long-term respiratory sequelae, such as asthma and other lung conditions [83,84,85] or allergies [86]. A recent study analyzed the DNA methylation profile in the blood of children under 2 years with RSV infection, which were monitored for at least 3 years for the development of asthma or recurrent wheezing [87]. Interestingly, at the acute phase of the disease, they identified over 5000 sites with different methylation levels between study participants who completely recovered and those who later developed asthma or wheezing. They further generated a model that could predict the development of these persistent symptoms with very high accuracy using only 3 of these features. The results from this study strongly support the hypothesis that the development of long-term respiratory symptoms by RSV is associated with the alteration of DNA methylation patterns in immune cells.

The concept of biological age, reflecting the functional status of various physiological systems, has gained prominence in recent years. Epigenetic clocks, which measure DNA methylation patterns associated with aging, offer a unique perspective on biological age. Recent advancements have led to the emergence of epigenetic biological age as a novel and insightful metric to gauge the overall health of an individual and the potential implications for long-term health [88,89,90,91]. It is well described that Human Immunodeficiency Virus (HIV) infection accelerates aging according to these epigenetic clocks based on DNA methylation patterns [92,93]. In addition, healthy adults with cytomegalovirus (CMV) seropositivity were found to show an older epigenetic age than those that were CMV seronegative [94]. Interestingly, acute-severe infection by SARS-CoV-2, but not by IAV, was also found to induce DNA methylation modifications in patients that resulted in an older epigenetic age [95]. Therefore, not only chronic virus infection but also transient infection might have an effect on the epigenetic signatures associated with aging. How the accumulation of these epigenetic modifications shapes our innate immune system and what effect it has on aging is not known. The emerging “inflammageing” concept [96], characterized by an unbalanced inflammation in the absence of stimuli, might reflect the accumulation of epigenetic changes induced by previous exposures [97], including virus infections.

The role of lncRNA in the epigenetic regulation of respiratory viral infection is also increasingly evident in human studies. A study that analyzed samples from patients diagnosed with SARS-CoV-2 and IAV infections identified hundreds of lncRNA with different levels in both types of infection as compared to negative controls. Among those, they identified one lncRNA named CHROMR, which is involved in the metabolism of lipids and is associated with changes in histone acetylation at regulatory regions of ISGs. CHROMR was shown to be important for the restriction of viral infection in macrophages [98].

**Table 1:** Summary of findings from human studies on the induction of epigenetic changes in blood from subjects infected by SARS-CoV-2, IAV, and RSV.

Virus	Sample Type	Type of Analysis	Description of Epigenetic Changes	References
SARS-CoV-2	Peripheral whole blood	DNA methylation arrays Bulk RNA-seq	Persistent changes with a high proportion of hypomethylated sites in ISGs. Overlap with SLE and MS.	[78]
	PBMCs	Single-cell ATAC-seq	Changes in CD14^+^ and CD16^+^ monocytes, consistent with trained immunity. Epigenetic changes also identified in adaptive immune cells.	[79]
	PBMCs	Single-cell ATAC-seq Single-cell RNA-seq DNA methylation arrays	Minor epigenetic differences, mapped to monocytes and CD4^+^ T cells.	[80]
	Monocytes and HSPC (enriched from PBMCs)	Paired single-cell RNA-seq/ATAC-seq Bulk ATAC-seq	Epigenetic changes in monocytes and HSPC up to 1 year after infection, characterized by inflammatory signatures and increased myelopoiesis.	[81]
	Peripheral whole blood	Bulk RNA-seq	930 differentially expressed LncRNA in COVID-19 patients vs. controls	[98]
IAV	Peripheral whole blood	Bulk RNA-seq	340 differentially expressed LncRNA in IAV patients vs. controls	[98]
RSV	Peripheral whole blood	DNA methylation arrays	>5000 DMS in RSV-infected children that developed asthma or wheezing versus those with RSV infection but no long-term conditions.	[87]

PBMCs: Peripheral Blood Mononuclear Cells. SLE: Systemic Lupus Erythematosus. MS: Multiple Sclerosis. HSPC: Hematopoietic Stem and Progenitor Cells.

### 3.2. Evidence of Epigenetic Immune Regulation after Acute Virus Infection from In Vitro and Animal Models

Human studies indicate that viruses induce changes in our epigenetic machinery, probably because of the initiation of the innate and adaptive immune response to defend the organism and defeat the virus. Some of these epigenetic changes might be transient and revert soon after the infection is cleared, while other changes could last a longer time and potentially lead to immune memory, tolerance, or immune dysregulation. Here, we discuss the insights that animal models and in vitro models have provided on the effects of SARS-CoV-2, IAV, and RSV infection in the epigenetic remodeling of the innate immune system.

One of the ways that infection by respiratory viruses influences the epigenetic landscape in blood immune cells or infected cells could be through promoting the upregulation or downregulation of epigenetically involved enzymes (Table 2), such as DNMTs or HDACs. For example, a study characterized the effect of IAV infection in DNA methylation in PBMCs from human healthy donors exposed in vitro to either an H1N1 or an H5N1 IAV strain [99]. Interestingly, both strains upregulated DNMT1, DNMT3a, DNMT3b, TET1 and TET3; however, only H1N1 induced the expression of TET2 and significant levels of total demethylation. H1N1 also reduced the expression of UHRF1, a key epigenetic regulator that binds to DNA and recruits DNMT1 to promote methylation [99]. The authors found that the downregulation of UHRF1 promoted IFNB1 expression by demethylating one single CpG site in the IFNB1 promoter. Another study analyzed the expression of this group of enzymes in A549 cells, a lung epithelial cell line that supports IAV replication [100]. Contrary to what the previous study found in blood cells, they observed that viral infection resulted in the reduced expression of DNMT1, DNMT3a, and DNMT3b. In addition, they found that IAV infection altered the DNA methylation landscape, including changes in the promoters of multiple genes involved in virus–host interactions (e.g., OASL, IFITM3, and SIGLEC1). This study also found that changes in DNMT1 expression influenced levels of DNA methylation of the OASL promoter, altering its gene expression [100]. Another study showed similar downregulation of DNMT1, DNMT3a, and DNMT3b in human epithelial cells infected with SARS-CoV-2 in vitro [101]. IAV was also found to downregulate HDAC1 and HDAC2 expression in vitro, and a reduction in HDAC1 or HDAC2 expression is associated with increased virus replication [102,103]. While these studies demonstrate that infection might modify the expression of the main enzymes controlling DNA methylation or histone modifications, the mechanisms leading to these changes are still not well understood.

IAV infection has been shown to induce histone modifications. A549 cells infected with IAV showed a decrease in histone acetylation 8 h post-infection, which could contribute to impaired cellular transcription [104]. Additionally, an increase in H3K79 methylation was observed. The inhibition or silencing of the expression of the enzyme responsible for this methylation, Dot1L, led to increased replication of the virus. Furthermore, this study revealed a decrease in the antiviral response when Dot1L was downregulated, suggesting that the methylation of H3K79 plays a crucial role in controlling the antiviral signaling pathway. H3K79 methylation influenced the replication of other viruses that induce type I IFN production, highlighting its potential significance in the regulation of IFN-mediated signaling in the defense against viral pathogens [104]. A mouse study found that IAV infection increased the expression of the methyltransferase Setdb2 in myeloid cells in the lung, the expression of which is induced as a response to type I IFN signaling. Setdb2 tri-methylates H3K9, and this results in the reduced transcription of Mx1 and ISG15, in addition to a series of genes involved in inflammation, such as CCL2 [43]. RSV infection has also been found to alter the expression of the histone methylation machinery. In the mouse model, RSV upregulated the expression of Jmjd3 and Utx H3K27 demethylases in bone marrow-derived DCs and lung DCs [105].

In immune blood cells, the induction of epigenetic changes by viral infections are probably a result of paracrine signaling since cell types such as PBMCs are not the main cell targets for viruses such as SARS-CoV-2, IAV, or RSV for replication. However, in target cells, such as epithelial cells, the epigenetic changes could be due to a direct interaction of host proteins with viral proteins involved in immune evasion. Multiple viral proteins have been shown to interact with cellular proteins, interfering with the cellular epigenetic landscape (Table 3). A proteomics study identified interactions between SARS-CoV-2 proteins and several human proteins involved in epigenetic regulation. Among these, some interesting interactions were NSP5 with HDAC2 [106,107] and E protein with BRD2 and BRD4 [106,108]. The functional aspects of these interactions remain to be studied. Interestingly, a different study found that the protein encoded by ORF8 functioned as a histone mimic of H3, disrupting host cell epigenetic regulation and facilitating virus replication [109,110]. Proteins from SARS-CoV-2 variants could potentially differ in terms of their ability to interact with the epigenetics machinery, resulting in diverse mechanisms to exploit the host defenses (reviewed by [111]).

In the case of IAV, the non-structural (NS) 1 protein, a multifunctional protein with the ability to counteract the innate immune system, was shown to interact with DNMT3b and disassociate it from gene promoters of several key regulators of the JAK-STAT signaling pathway [112]. In addition, NS1 from H3N2 viruses was found to be an H3 mimic that suppressed the antiviral response [113]. Another protein from IAV, the accessory protein PA-X, which has endoribonuclease activity, inhibits type I IFN by degrading TET2 [114]. TET2 was found to regulate the expression of STAT1 and multiple IFN-stimulated genes (ISGs) through demethylation [114]. Furthermore, IAV proteins have been found to interact with chromatin remodelers as well. For example, PA and the viral polymerase complex interact with CHD6 [115,116], and infection induces CHD6 relocation to heterochromatin, indicating that this interaction could have important effects on chromatin remodeling and gene transcription. The NEP (NS2) protein of IAV was found to interact with CHD3, and this interaction is important in terms of promoting vRNPs nuclear export and efficient replication of the virus [117].

The NS1 protein of RSV was also shown to interact with the epigenetic machinery to modulate host gene transcription. RSV NS1 is associated with chromatin in infected cells and interacts with the Mediator complex [118]. Moreover, the NS1 protein from RSV interacts with the histone H2B in bronchial epithelial cells and induces H2B ubiquitination [119]. The downstream effects of the NS1-induced H2B ubiquitination have not been studied.

**Table 3:** Summary of known interactions of viral proteins with cellular proteins of the epigenetics machinery.

Virus	Viral Protein	Interacting Host Molecule/Complex	Consequences	References
SARS-CoV-2	NSP5	HDAC2	Unknown.	[106,107]
	E	BDR2 and BDR4	Unknown.	[106,108]
	ORF8	Chromatin-associated proteins (H3 mimic)	Promotes chromatin compaction. Facilitates virus replication.	[109]
IAV	NS1 (H3N2)	Human PAF1C (H3 mimic)	Suppression of antiviral response.	[113]
	NS1	DNMT3b	Disassociation of DNMT3b from gene promoters related to JAK-STAT signaling.	[112]
	PA-X	TET2 mRNA	Degradation of TET2 mRNA, leading to changes in STAT1 expression and downstream ISG expression. Facilitates replication.	[114]
	PA/viral polymerase complex	CHD6	Potential effects on chromatin remodeling.	[115,116]
	NEP	CHD3	Promote vRNA export and virus replication.	[117]
RSV	NS1	Mediator complex/chromatin	Potential modulation of the antiviral response.	[118]
	NS1	H2BD	Induces H2B ubiquitination.	[119]

Overall, these in vitro and animal studies collectively support a broad effect of viral infections in the induction of epigenetic changes through multiple mechanisms, including DNA methylation and histone modifications. These modifications play a crucial role in modulating the expression of immune-related genes, thereby influencing the host response to viral infections.

## 4. Concluding Remarks

**Figure 1:**
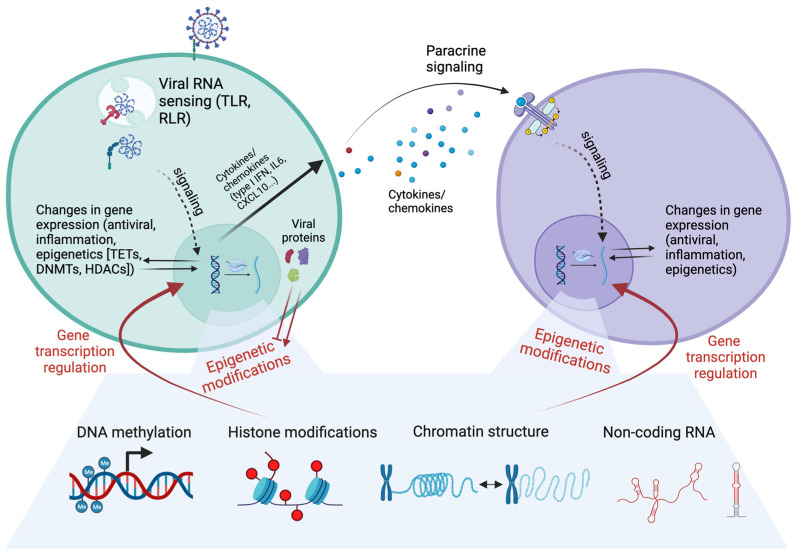
Overview of the potential mechanisms of epigenetic regulation of the innate immune system induced by viruses such as SARS-CoV-2, IAV or RSV. Upon virus internalization, cells sense the virus components (PAMPs) by multiple PRRs such as TLRs and cytosolic receptors (RIG-I like receptors [RLRs]). Downstream signaling events lead to changes in transcription and epigenetic modifications. Those epigenetic modifications have an effect on the further modulation of gene transcription. Viral proteins in the infected cell might have roles in inhibiting or inducing epigenetic modifications through direct protein-protein interactions. Cytokines produced by the infected cell bind receptors in neighboring cells, inducing activation of signaling cascades that also induce changes in transcription and epigenetic modifications. Figure created using Biorender.

Acute viral infection induces a wide spectrum of epigenetic modifications related to the regulation of the innate immune system. DNA methylation, histone modifications, and other epigenetic components involved in chromatin remodeling regulate the expression of key immune genes, influencing the balance between antiviral defenses and immune tolerance. In addition, viral infections alter the epigenetic profile of blood immune cells by multiple mechanisms, which also influence future responses to innate immune stimuli. Mechanisms of epigenetic modulation by virus infection include virus sensing through PRRs and subsequent signaling events, altered expression of proteins involved in the epigenetic machinery, paracrine effects of cytokine signaling in infected and neighboring cells, and direct interaction of virus proteins with host epigenetic proteins (Figure 1).

There are some important questions in this field that remain to be elucidated. One key aspect that needs to be better understood is the potential consequences of epigenetic remodeling of the immune system by respiratory virus infections and potential long-term health effects, such as PASC in the case of SARS-CoV-2 or higher predisposition to chronic respiratory diseases in the case of RSV. In connection with this, it is not clear if the accumulation of these acute exposures to viral infections could increase the predisposition to future immune-related or auto-immune disorders. In addition, we need to achieve better insights into the cell-specificity of the epigenetic regulations. Monocytes have been broadly studied, given their well-described role in trained immunity. However, epigenetic regulation of other important innate immune cells, such as dendritic cells, macrophages, NK cells, or neutrophils, remains to be understood. The longevity of the epigenetic modification of immune responses for the different viruses, or association with disease severity, is not well defined. In addition, the impact of virus-induced epigenetic modification in the regulation of the immune response during subsequent exposures, such as infections or vaccination, is not known. Another open question is how the modulation of the innate immune response through epigenetic mechanisms might have consequences in the magnitude and quality of the adaptive immune response.

In conclusion, the field holds intriguing questions that demand elucidation. The identification of specific epigenetic modifications associated with altered immune responses opens avenues for therapeutic interventions. The crosstalk between epigenetic modifications and immune responses is a critical determinant of the outcomes of viral infections in humans. Therefore, elucidating the intricacies of this interplay provides a foundation for developing targeted therapeutic strategies that leverage the host’s epigenetic machinery to improve clinical outcomes. Targeting these modifications could potentially enhance antiviral immunity or improve the efficacy of vaccines or antiviral drugs. Continued research in this field holds promise for advancing our understanding of host-virus interactions and informing the development of innovative antiviral therapies.

## Figures and Tables

**Table 2:** Summary of studies reporting effects of viral infection in the epigenetic landscape using experimental models (in vitro and in vivo).

Virus	Experimental System	Main Findings on Epigenetic Modulation	References
IAV	In vitro, PBMCs; Mouse model	Upregulation of DNMT1, DNMT3a, DNMT3b, TET1 and TET3 by H1N1 or H5N1; TET2 induced only by H1N1; UHFR1 expression reduced by H1N1. UHRF1 expression regulated IFN-beta expression by a mechanism involving a single-nucleotide methylation in IFN-beta promoter.	[99]
IAV	In vitro, A549 cells	Reduced expression of DNMT1, DNMT3a, and DNMT3b; altered DNA methylation affecting ISGs (OASL, IFITM3, SIGLEC1). 80 miRNA upregulated; miR-142-5p downregulates DNMT1.	[100]
IAV	In vitro, A549 cells	H1N1 downregulates HDAC1 and increases H3 acetylation; HDAC1 has an antiviral effect.	[102]
IAV	In vitro, A549 cells	H1N1 downregulates HDAC2; HDAC2 has an antiviral effect.	[103]
IAV	In vitro, A549 cells	H1N1 (A/Puerto Rico/8/34) decreases histone acetylation and increases H3K79 methylation. Inhibition of H3K79 methylation results in increased virus replication.	[104]
IAV	Mouse model	Increased expression methyltransferase Setdb2 in lung myeloid cells upon infection; increased H3K9me3 leading to reduced Mx1 and ISG15 expression.	[43]
RSV	Mouse model	Increased expression of H3K27 demethylases in bone marrow and lung dendritic cells.	[105]
SARS-CoV-2	In vitro, A549 and NHBE cells	Infection downregulates DNMT1, DNMT3a, and DNMT3b.	[101]

## Data Availability

No new data were created or analyzed in this study. Data sharing is not applicable to this article.
